# Acute and Chronic Resistance Training Differentially Modulate Myocardial Angiogenesis–Related Gene Expression in Wistar Rats

**DOI:** 10.1155/crp/2976039

**Published:** 2026-10-18

**Authors:** Putri Karisa, Nova Sylviana, Aziiz Mardanarian Rosdianto, Hanna Goenawan, Mas Rizky Anggun Adipurna Syamsunarno, Setiawan Setiawan

**Affiliations:** ^1^ Faculty of Medicine Padjadjaran University Bandung 40161 West Java Indonesia unpad.ac.id; ^2^ Cardiovascular and Metabolic Medicine Research Group Faculty of Medicine Padjadjaran University Bandung 40161 West Java Indonesia unpad.ac.id; ^3^ Department of Biomedical Sciences Faculty of Medicine Padjadjaran University Jatinangor 45363 West Java Indonesia unpad.ac.id; ^4^ Physiology Molecular Laboratory Biology Activity Division Central Laboratory Padjadjaran University Jatinangor 45363 West Java Indonesia unpad.ac.id; ^5^ Center of Sport Science, Wellness, and Longevity, Graduate School Padjadjaran University Bandung 40161 West Java Indonesia unpad.ac.id

**Keywords:** angiogenesis, exercise, myocardium, resistance training

## Abstract

Myocardial angiogenesis contributes to the maintenance of myocardial perfusion and cardiac function. Resistance training (RT) induces cardiovascular adaptations, but the myocardial transcriptional responses to acute RT (ART) and chronic RT (CRT) remain incompletely understood. This study compared the expression of angiogenesis‐ and metabolic adaptation–related genes in the left ventricle following a single RT bout and 8 weeks of repeated RT in healthy male Wistar rats. Twenty‐four rats were randomized to three groups: sedentary control (CON) (*n* = 8), ART (one ladder‐climbing session), or CRT (ladder‐climbing 5 days/week for 8 weeks). RT was performed on a 1‐m ladder inclined at 80° using progressive loads attached to the tail. Left‐ventricular tissue was collected 1 h after the exercise bout in ART and 1 h after the final training session in CRT. Compared to CON, ART showed significantly higher HIF‐1*α* and FSTL1 expression, whereas PGC‐1*α* and VEGF did not differ significantly. CRT also showed significantly higher HIF‐1*α* and FSTL1 expression than CON, together with significantly higher PGC‐1*α* and VEGF expression. No significant differences were observed between ART and CRT for any of the genes examined. These findings demonstrate distinct myocardial transcriptional profiles following ART versus CRT, characterized by an HIF‐1α‐ and FSTL1‐associated response after a single bout and a PGC‐1α‐ and VEGF‐associated response following CRT.

## 1. Introduction

Resistance training (RT) is increasingly recognized as an important stimulus for physiological cardiac remodeling in addition to its well‐established effects on skeletal muscle strength and metabolic health [1]. Regular physical activity is consistently associated with reduced cardiovascular disease incidence and mortality, and current international guidelines recommend muscle‐strengthening exercise as an integral component of exercise prescription for cardiovascular health [2, 3]. The cardiac response to RT differs fundamentally from that of aerobic training. During resistance exercise, cardiac output primarily increases through elevations in heart rate, whereas changes in stroke volume are generally smaller than those observed during aerobic exercise [4]. Consequently, RT is characterized by transient increases in arterial pressure, myocardial wall stress, and afterload, creating a distinct hemodynamic stimulus for cardiac remodeling [5]. Although aerobic exercise has traditionally been the focus of cardiovascular exercise research, accumulating evidence indicates that RT also induces cardiac remodeling through distinct mechanical and metabolic stimuli that may engage different molecular signaling pathways [6].

Hemodynamic changes in RT result in a pattern of cardiac remodeling driven by pressure overload, leading to concentric hypertrophy [7]. Physiological cardiac hypertrophy induced by RT is typically accompanied by little or no fibrosis and by proportional increases in myocardial capillary density [7, 8]. The maintenance of this physiological remodeling depends on coordinated growth of both cardiomyocytes and the coronary microvasculature, ensuring adequate oxygen delivery to meet the increased metabolic demands of the hypertrophied myocardium. Consequently, proportional expansion of the coronary microvasculature distinguishes physiological hypertrophy from pathological remodeling, in which capillary rarefaction contributes to impaired myocardial perfusion [9]. Angiogenesis is a multistep process involving the formation of new capillaries from preexisting vessels and is regulated by a complex interaction of proangiogenic and antiangiogenic factors [10]. Adequate myocardial vascular adaptation is important for maintaining tissue perfusion and matching oxygen and nutrient delivery with myocardial metabolic demand [11]. An 8‐week study in humans showed that RT positively modulates endothelial progenitor cells (CD34+ and VEGFR2+) and angiogenesis‐related genes (hypoxia‐inducible factor‐1*α* [HIF‐1*α*], vascular endothelial growth factor [VEGF], and SDF‐1) [12]. Other studies have shown that both acute and 4‐week RT significantly increase VEGF expression under normoxic and hypoxic conditions, promoting angiogenic responses [13, 14]. Further evidence from animal models of myocardial infarction indicates that RT enhances vascularization compared to controls (CONs) [15, 16].

Angiogenic adaptation is regulated through multiple interconnected signaling pathways. Traditionally, HIF‐1*α* has been regarded as the principal oxygen‐sensitive regulator of angiogenesis because it stimulates transcription of VEGF during reduced tissue oxygen availability [17]. However, accumulating evidence indicates that physiological exercise can also activate VEGF through hypoxia‐independent mechanisms. This distinction is particularly relevant because physiological exercise may increase energetic demand without producing sustained tissue hypoxia. In particular, peroxisome proliferator–activated receptor gamma coactivator‐1*α* (PGC‐1*α*), a master regulator of mitochondrial biogenesis and oxidative metabolism, promotes VEGF expression independently of canonical HIF signaling in response to increased energetic demand [15, 18–20]. These observations suggest that exercise‐induced angiogenesis may result from the integration of oxygen‐dependent and metabolic signaling rather than hypoxia alone. In addition to metabolic regulators, cardiokines have emerged as important modulators of vascular adaptation. Among these, FSTL1 has been shown to promote vascular growth, endothelial cell survival, and tissue repair through activation of Akt/ endothelial nitric oxide (NO) synthase (eNOS) signaling and interactions with VEGF‐mediated pathways [21]. In experimental myocardial infarction, resistance exercise increases myocardial FSTL1 expression and is accompanied by enhanced angiogenic remodeling, suggesting that FSTL1 may represent an additional regulator of exercise‐induced cardiac vascular adaptation [15, 22].

Collectively, existing evidence demonstrates that exercise regulates angiogenesis‐related signaling in the myocardium. However, most available studies have investigated aerobic exercise [18] or pathological models such as myocardial infarction [15, 22, 23] and aging [24], whereas considerably less is known about physiological adaptations induced by RT in the healthy heart. Furthermore, although HIF‐1*α* is traditionally viewed as the central regulator of exercise‐induced angiogenesis, the extent to which RT in the healthy myocardium produces sufficient or sustained myocardial hypoxia to maintain HIF‐1*α* activation remains uncertain. Experimental deletion of HIF‐1*α* in skeletal muscle induces adaptations resembling endurance training, including increased capillary density through constitutive activation of AMPK‐dependent metabolic signaling, indicating that angiogenesis can be preserved despite the absence of canonical HIF signaling [25]. These findings raise the possibility that repeated RT may differentially regulate hypoxia‐associated and metabolic signaling pathways during physiological myocardial adaptation, although the temporal patterns of these responses remain unclear.

Although previous studies have investigated molecular responses to acute and chronic exercise, it remains unclear whether a single resistance exercise bout and repeated RT are associated with distinct myocardial transcriptional responses. In particular, the relative responses of hypoxia‐associated, metabolic, and angiogenesis‐related genes following acute versus repeated resistance exercise have not been fully characterized. Therefore, the present study examined the expression of HIF‐1*α*, PGC‐1*α*, FSTL1, and VEGF in the left ventricle of healthy male Wistar rats following a single resistance exercise bout or an 8‐week resistance‐training regimen. We hypothesized that a single bout and repeated RT would produce distinct myocardial expression profiles of these angiogenesis‐related genes.

## 2. Methods

### 2.1. Animal Model and Experimental Design

Male Wistar rats aged 8 weeks (180–200 g) were used in this study. At this age, rats are considered young adults that have reached sexual maturity while continuing physiological maturation, making them suitable for investigating exercise‐induced cardiac adaptations with minimal influence of aging [26]. After a 1‐week acclimation period, animals were randomly assigned using a computer‐generated randomization sequence to one of three groups: sedentary CON (*n* = 8), acute RT (ART) (*n* = 8), or chronic RT (CRT) for 8 weeks (*n* = 8). Group allocation was performed by an investigator who was not involved in the molecular analyses. An earlier version of this work has been made publicly available as a preprint on Research Square [27].

Sample size was determined using Mead’s resource equation for in vivo studies with one‐way ANOVA [28]. Based on this approach, a minimum of six rats per group was required. To account for potential attrition during the intervention, two additional animals were included in each group, resulting in a total sample size of 24 rats (eight per group).

All experimental procedures were approved by the Research Ethics Committee of Universitas Padjadjaran (No. 1030/UN6.KEP/EC/2024) and conducted in accordance with relevant institutional guidelines and the ARRIVE guidelines. Rats were housed four per cage (45 × 30 × 25 cm) under controlled environmental conditions (22 ± 2°C, 50%–60% humidity, 12‐h light/12‐h dark cycle) at the Laboratory of Universitas Padjadjaran (Bandung, Indonesia). Standard laboratory chow (Prospets; 302.94 kcal/100 g) and water were provided *ad libitum*. Animals showing injury, severe weight loss, feeding difficulties, inactivity, illness (e.g., infection, fur loss, aggression, or head tilt), or failure to complete the exercise protocol during familiarization were excluded. No animals met the exclusion criteria.

### 2.2. Exercise Protocol

RT was performed by stair climbing by tying weights to the tail of the rat. Preadaptation training was conducted using a ladder for laboratory animals (length 1 m; 80° slope, with a distance of 2 cm between steps) adapted from Lee et al., which has been shown to cause muscle hypertrophy in rats after exercise [29]. The selection of ladder climbing as a research method is based on the efficiency of RT, which can involve many muscle groups (including the heart), and its use has been proven in various related studies [30]. A load was attached to the base of the tail with hypoallergenic adhesive tape and a nonelastic cord, ensuring that the weight could not be removed by the animal. One full climb from the bottom to the top of the ladder was defined as one repetition.

To familiarize the rats with the ladder, a 1‐week acclimation process was implemented without the load. Acclimation was performed in three ways. First, rats were placed at the top of the ladder, approximately 35 cm from the box, to create a sense of security in the ladder environment. In the second method, the rats were placed from the direction of the stairs, approximately 55 cm from the top box, and then allowed to climb the stairs. In the third method, the rats were placed at the bottom of the stairs, about 110 cm from the top box, and then allowed to climb the stairs [31].

The acute resistance exercise protocol was adapted from Begue et al., whereas the CRT protocol was modified from previously described ladder‐climbing protocols [32]. In the ART group, rats were given one training session with weights ranging from 25%, 50%, and 75% of body weight, each comprising four repetitions, followed by one set at 100% BW comprising six repetitions. The rats were allowed 2 min to rest for each repetition.

In the CRT group, rats trained 5 days per week for 8 consecutive weeks. Each session started with a load of 50% BW. If a rat successfully completed 10 repetitions in a session, the load for the next session was increased by 10% of the previous load (for example, from 50% to 60% BW), up to the maximal load tolerated. The maximal load was defined as the heaviest weight that the rat could lift while completing at least one successful climb. All CRT sessions consisted of 10 repetitions with 2‐min rest intervals between repetitions. Body weight was recorded daily and used to adjust the absolute load. The same experienced investigator supervised all exercise sessions to ensure consistent application of the protocol [15, 16, 31–33]. All exercise sessions were conducted at the same time of day to minimize circadian variation. The CON animals should have undergone equivalent handling/environmental exposure without resistance exercise.

### 2.3. Tissue Collection and RNA Extraction

At the end of the experimental period, rats in the ART group were euthanized for tissue collection 1 h after completion of the single resistance exercise bout, whereas rats in the CRT group were euthanized 1 h after completion of the final resistance‐training session. The same postexercise sampling interval was applied to both exercise groups to facilitate comparison of early myocardial transcriptional responses following acute and repeated resistance exercise. The CON animals were euthanized at the corresponding time point under the same experimental conditions. Euthanasia was performed under deep anesthesia induced by intraperitoneal ketamine (45 mg/kg) and xylazine (5 mg/kg), followed by exsanguination via cardiac puncture and bilateral thoracotomy. Adequate anesthesia was confirmed by the absence of the pedal withdrawal reflex before thoracotomy, and death was confirmed by cessation of heartbeat. Ketamine–xylazine anesthesia was selected because it provides stable surgical anesthesia and is widely used for terminal tissue collection in rodents [34]. Left ventricular tissue was collected for gene expression analysis. Total RNA was extracted using GENEzol reagent according to the manufacturer’s instructions (Geneaid, Taipei, Taiwan). RNA concentration and purity were assessed spectrophotometrically by measuring absorbance at 260 and 280 nm, and only samples with A260/A280 ratios between 1.8 and 2.0 were included for downstream analysis. The extracted RNA was subsequently used for one‐step quantitative reverse transcription polymerase chain reaction (RT‐qPCR). The primer sequences for FSTL1, HIF‐1*α*, PGC‐1*α*, VEGF, and glyceraldehyde‐3‐phosphate dehydrogenase (GAPDH) are presented in Table 1. The four genes were prespecified angiogenesis‐related molecular outcomes.

**TABLE 1:** Primer sequences.

Genes	Annealing temperature	Forward primer 5′‐3′	Reverse primer 5′‐3′
FSTL1	62	GACTGCAATCGCTGTGTCTGTTC	TCCTGGGCATATCTCGTCATCTC
HIF‐1*α*	56	CTCCCATACAAGGCAGCAGAAA	CAAAACAACCAACAGAAACGAAAC
PGC‐1*α*	62	CGCACAACTCAGCAAGTCCTC	CCTTGCTGGCCTCCAAAGTCTC
VEGF	58	CTGGACCCTGGCTTTACTGC	AATTGGACGGCAATAGCTGCG
GAPDH	58	GTTACCAGGGCTGCCTTCTC	GATGGTGATGGGTTTCCCGT

### 2.4. Real‐Time PCR Analysis

Gene expression levels of FSTL1, HIF‐1*α*, PGC‐1*α*, and VEGF were quantified using RT‐qPCR, with GAPDH as the reference gene for normalization. Each 10‐μL reaction contained 5 μL of 2 × SensiFAST SYBR Lo‐ROX mix, 0.4 μL each of forward and reverse primers, 0.2 μL RNase inhibitor, 0.1 μL reverse transcriptase, 2 μL total RNA, and 1.9 μL nuclease‐free water (SensiFAST SYBR Lo‐ROX One‐Step Kit, Bioline, USA) [35]. RT‐qPCR was performed using an AriaMx Real‐Time PCR System (Agilent Technologies, USA). The thermal cycling conditions consisted of reverse transcription at 45°C for 10 min, initial denaturation at 95°C for 2 min, followed by 40 cycles of denaturation at 95°C for 5 s and annealing/extension at the gene‐specific annealing temperature (56°C–62°C; Table 1) for 20 s. A melting‐curve analysis (65°C–95°C with 0.5°C increments) was performed after amplification to verify the specificity of each amplicon. GAPDH was used as the reference gene, and its expression remained stable across experimental groups, as demonstrated by comparable Cq values (Supporting Figure S1). Primer sequences for FSTL1, HIF‐1*α*, PGC‐1*α*, VEGF, and GAPDH are listed in Table 1. All reactions were performed in technical duplicate, and the mean Cq value of the technical replicates was used as the biological replicate value for each animal. Relative gene expression was calculated using the 2^‐ΔΔCt method with GAPDH as the reference gene [36]. The RT‐qPCR experiments were conducted and reported in accordance with the *Minimum Information for Publication of Quantitative Real-Time PCR Experiments* (MIQE) guidelines where applicable.

### 2.5. Statistical Analysis

Statistical analysis was conducted using the SPSS software for data analysis (Version 26.0) and GraphPad Prism 9 for visualization. Normality was assessed using the Shapiro–Wilk test. Homogeneity of variance was evaluated using Levene’s test. Normally distributed data are presented as mean ± SEM. Differences among groups were analyzed using one‐way ANOVA followed by Tukey’s multiple‐comparison test. Exact *p* values are reported throughout the Results where appropriate. Statistical significance was accepted at *p* < 0.05. Effect sizes (*η* ^2^ or Cohen’s d) and 95% confidence intervals were calculated where appropriate.

## 3. Results

### 3.1. Body and Heart Weight

Body and heart weight are presented in Table 2. Final body weight did not differ significantly among the CON, ART, and CRT groups (*p* = 0.112). Similarly, no significant between‐group differences were observed in heart weight (*p* = 0.855) or the heart weight‐to‐body weight ratio (*p* = 0.689). Mean final body weight was 362.8 ± 10.65 g, 346.3 ± 5.88 g, and 333.0 ± 11.2 g in the CON, ART, and CRT groups, respectively. Heart weight was 1.00 ± 0.06 g, 1.00 ± 0.03 g, and 0.97 ± 0.05 g, while the heart weight‐to‐body weight ratio was 2.77 ± 0.17, 2.89 ± 0.14, and 2.92 ± 0.04 mg/g, respectively. No gross differences in heart weight or heart‐to‐body weight ratio were detected.

**TABLE 2:** Body and heart weight.

Variable	Control (*n* = 8)	ART (*n* = 8)	CRT (*n* = 8)	*p* value
Final body weight (g)	362.8 ± 10.65	346.3 ± 5.88	333 ± 11.2	0.112
Heart weight (g)	1 ± 0.06	1 ± 0.03	0.97 ± 0.05	0.855
Heart weight/body weight ratio (mg/g)	2.77 ± 0.17	2.89 ± 0.14	2.92 ± 0.04	0.689

*Note:* Data are presented as mean ± SEM.

Abbreviations: ART, acute resistance training; CRT, chronic resistance training.

### 3.2. RT Load

RT load increased progressively during both ART and CRT (Figure 1). During the ART session, load increased from 25% to 100% of BW across the prescribed loading steps (Figure 1A). Resistance‐training load increased progressively throughout the 8‐week CRT protocol, reflecting the progressive overload applied during the intervention from approximately 130 g in Week 1 to approximately 630 g in Week 8 (Figure 1B). Compared to Week 1, load was significantly higher from Week 2 onward (*p* < 0.05), with each subsequent week also showing a significant increase compared wto the preceding week (#*p* < 0.05).

**FIGURE 1:**
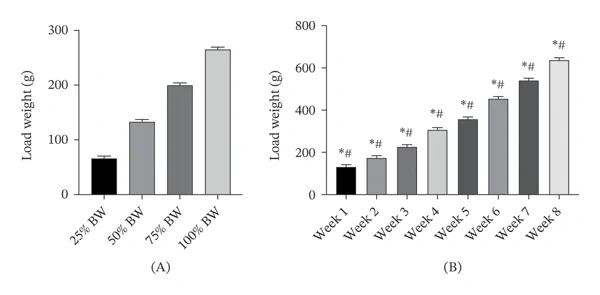
Resistance training load during acute and chronic resistance training. (A) Resistance training load during a single acute resistance‐training (ART) session. Rats performed four repetitions at 25%, 50%, and 75% of body weight (BW), followed by six repetitions at 100% BW. (B) Resistance training load during the 8‐week chronic resistance‐training (CRT) protocol. Training load was progressively increased according to each rat’s performance. Data are presented as mean ± SEM. *p* < 0.05 versus Week 1; #*p* < 0.05 versus the preceding week.

### 3.3. Impact of RT on Cardiac Angiogenesis–Related Genes

RT was associated with significant differences in the myocardial expression of hypoxia‐, angiogenesis‐, and metabolic adaptation–related genes (Figure 2). HIF‐1*α* expression differed significantly among groups (*F*(2,21) = 7.041, *p* < 0.01, *η* ^2^ = 0.401, 95% CI: 0.056–0.590), with post hoc analysis showing significantly higher expression in the ART group than in the CON group (mean difference = 0.375, 95% CI: 0.0986–0.6514, *p* < 0.01) and in the CRT group than in the CON group (mean difference = 0.305, 95% CI: 0.0286–0.5814, *p* < 0.05), whereas ART and CRT did not differ significantly (mean difference = −0.070, 95% CI: −0.3464–0.2064, *p* > 0.05). PGC‐1*α* expression also differed significantly among groups (*F*(2,21) = 4.632, *p* < 0.05, *η* ^2^ = 0.306, 95% CI: 0.004–0.516), with significantly higher expression in the CRT group than in the CON group (mean difference = 1.526, 95% CI: 0.2130–2.8395, *p* < 0.05), while no significant differences were observed between the CON and ART groups (mean difference = 0.610, 95% CI: −0.7032–1.9232, *p* > 0.05) or between ART and CRT (mean difference = −0.916, 95% CI: −2.2295–0.3970, *p* > 0.05). FSTL1 expression showed a significant between‐group difference (*F*(2,21) = 10.121, *p* < 0.001, *η* ^2^ = 0.491, 95% CI: 0.130–0.656), with higher expression in the ART group than in the CON group (mean difference = 3.294, 95% CI: 1.3085–5.2790, *p* < 0.001) and in the CRT group than in the CON group (mean difference = 2.486, 95% CI: 0.5010–4.4715, *p* < 0.05), whereas ART and CRT did not differ significantly (mean difference = 0.808, 95% CI: −1.1777–2.7927, *p* > 0.05). VEGF expression also differed significantly among groups (*F*(2,21) = 5.144, *p* < 0.05, *η* ^2^ = 0.329, 95% CI: 0.014–0.534), with significantly higher expression in the CRT group than in the CON group (mean difference = 0.614, 95% CI: 0.1006–1.1269, *p* < 0.05), whereas no significant differences were observed between the CON and ART groups (mean difference = 0.440, 95% CI: −0.0732–0.9532, *p* > 0.05) or between ART and CRT (mean difference = 0.174, 95% CI: −0.3394–0.6869, *p* > 0.05).

**FIGURE 2:**
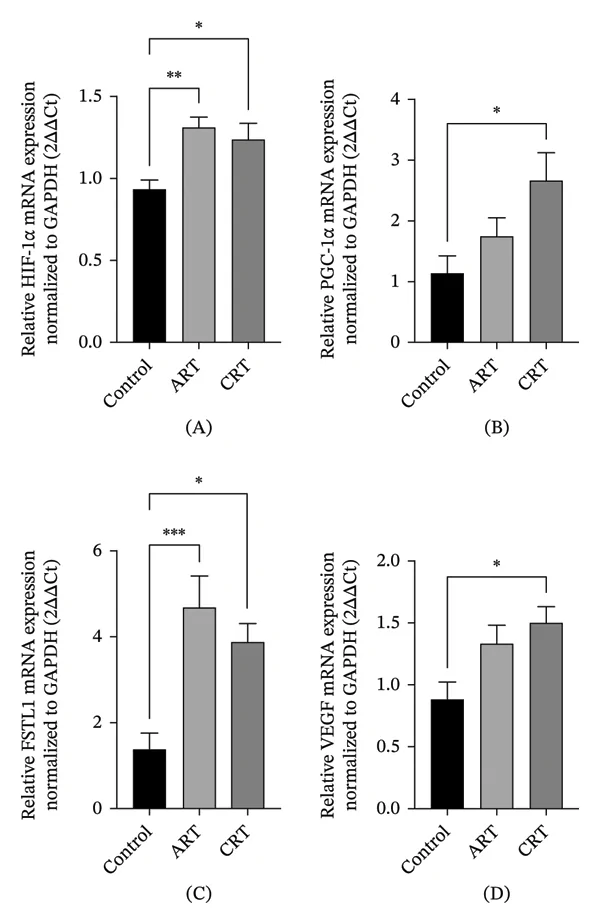
Cardiac gene expression of angiogenesis‐related regulators in control (CON), acute resistance training (ART), and chronic resistance training (CRT) groups. Panels show mRNA levels of (A) HIF‐1*α*, (B) PGC‐1*α*, (C) FSTL1, and (D) VEGF in left ventricular tissue. Data are presented as mean ± SEM (*n* = 8 per group). #*p* < 0.05, overall group effect by one‐way ANOVA; ^∗^ *p* < 0.05; ^∗∗^ *p* < 0.01; ^∗∗∗^ *p* < 0.001 vs. CON (Tukey’s post hoc test).

## 4. Discussion

The present study examined the effects of ART and CRT on myocardial genes related to angiogenesis and metabolic adaptation in healthy rats. RT altered the expression of several genes, with both shared and training‐related responses observed between the two exercise conditions. HIF‐1*α* mRNA expression was significantly higher in both ART and CRT compared to the CON group, while no significant difference was observed between ART and CRT. FSTL1 showed a similar pattern, with significantly higher expression in both training groups than in the CON group. In contrast, PGC‐1*α* and VEGF expression were significantly higher in CRT than in the CON group, whereas neither differed significantly between ART and CON or between ART and CRT. These findings suggest that RT induces an early myocardial transcriptional response involving HIF‐1*α* and FSTL1, while repeated training may additionally enhance metabolic and angiogenic signaling involving PGC‐1*α* and VEGF.

The higher HIF‐1*α* expression observed after both ART and CRT may reflect the increased metabolic and oxygen demands associated with resistance exercise. HIF‐1*α* is a central regulator of cellular responses to changes in oxygen availability and metabolic stress. Under normoxic conditions, HIF‐1*α* is regulated by oxygen‐dependent hydroxylation involving prolyl hydroxylase domain (PHD) enzymes and factor‐inhibiting HIF (FIH), which facilitates its degradation [37, 38]. Previous studies have reported greater HIF‐1*α* responses following acute exercise compared to chronic exercise, potentially reflecting adaptations to repeated exercise, including improved oxygen delivery and utilization [18, 37–39]. Acute metabolic or oxygen stress may also reduce hydroxylase activity through changes in oxygen availability and mitochondrial reactive oxygen species (ROS), thereby promoting HIF‐1*α* signaling [40–42]. Recent findings in an aerobic exercise model show an increase in HIF‐1*α* in the acute group, followed by a decrease in PHD and FIH, which are HIF inhibitors. This indicates that, during the acute phase, HIF inhibitors have not yet been induced, resulting in high levels of HIF‐1*α* [27]. However, because HIF‐1*α* expression was elevated in both ART and CRT in the present study, our findings do not support an acute‐specific HIF‐1*α* response. Instead, they suggest that HIF‐1*α* transcription remains responsive to the exercise bout even after chronic training. Importantly, this interpretation should remain cautious because HIF‐1*α* activity is primarily regulated through protein stability, degradation, and subcellular localization rather than mRNA expression alone.

Exercise‐induced vascular responses are also influenced by endothelial and hemodynamic signaling. Exercise can enhance NO‐mediated vasodilation through eNOS, contributing to vascular reactivity and tissue perfusion [43, 44]. eNOS activity may also interact with PI3K/Akt signaling and influence HIF‐1α–related responses [45, 46]. Although these pathways may contribute to the myocardial response to resistance exercise, the present study did not directly measure NO, eNOS activity, or Endothelin‐1 (ET‐1). Therefore, these mechanisms should be considered possible contributors rather than direct explanations for the observed HIF‐1*α* response. Previous evidence indicates that exercise‐induced coronary adaptation involves coordinated changes in vascular structure, vasomotor function, and coronary transport capacity [19, 24, 47, 48].

The increase in HIF‐1*α* expression may be relevant to the regulation of downstream angiogenic signaling. HIF‐1*α* can promote the transcription of hypoxia‐responsive genes, including VEGF, following changes in oxygen availability and metabolic stress [49, 50]. However, VEGF was significantly higher in CRT than in the CON group, whereas the ART group did not differ significantly from the CON group. Moreover, there was no significant difference in VEGF expression between ART and CRT. Thus, the present findings do not indicate a simple direct relationship in which increased HIF‐1*α* expression immediately results in increased VEGF expression after acute exercise. Instead, VEGF expression may be influenced by additional regulatory pathways, particularly after repeated training. Because the present study measured HIF‐1*α* and VEGF mRNA rather than protein abundance or transcriptional activity, these findings should not be interpreted as direct evidence of HIF‐1α–mediated VEGF activation.

The significant increase in VEGF expression in CRT may indicate an enhanced transcriptional environment that supports vascular adaptation following repeated resistance exercise. VEGF is an important regulator of endothelial proliferation, migration, and vascular remodeling [51, 52], while repeated exercise may promote structural and functional changes in the cardiovascular system, including adaptations in coronary vascular capacity and capillary exchange [51, 53]. Nevertheless, increased VEGF mRNA alone cannot establish that angiogenesis occurred. The present study did not assess VEGF protein, endothelial proliferation, or myocardial capillary density. Therefore, the observed VEGF elevation should be interpreted as a potential molecular signal supporting angiogenic adaptation rather than direct evidence of increased myocardial angiogenesis.

Interestingly, the increase in VEGF following CRT occurred together with higher PGC‐1*α* expression. PGC‐1*α* is an important transcriptional coactivator involved in mitochondrial biogenesis, oxidative metabolism, and exercise adaptation 61. Its regulation during exercise involves several upstream signals, including AMPK, ROS, NO, and calcium‐dependent pathways [54, 55]. Previous studies have also shown that PGC‐1*α* contributes to exercise‐induced angiogenesis through regulation of VEGF [18, 19, 56, 57]. In particular, PGC‐1*α* can promote VEGF expression through an ERRα‐dependent pathway, providing a mechanism that may complement canonical HIF signaling [19, 58]. The concurrent increase in PGC‐1*α* and VEGF observed in the CRT group is therefore consistent with the possibility that repeated RT enhances metabolic–angiogenic signaling through PGC‐1α–associated mechanisms. However, because PGC‐1*α* protein activity and ERRα signaling were not directly assessed, this proposed mechanism remains inferential.

The increase in FSTL1 expression in both ART and CRT provides another potential component of the myocardial response to resistance exercise. FSTL1 has been described as a cardioprotective and proangiogenic factor that can influence endothelial function through Akt/eNOS signaling [59–61]. Experimental studies have shown that FSTL1 can increase eNOS phosphorylation and promote endothelial migration and network formation, while inhibition of Akt or eNOS can attenuate these effects [61]. Thus, the increased FSTL1 expression observed in the present study may represent an exercise‐responsive signal involved in vascular regulation. Because FSTL1 expression was increased in both ART and CRT, this response may be related more directly to the exercise stimulus than to chronic training adaptation. Nevertheless, protein‐level FSTL1 and downstream Akt/eNOS activity were not measured, so the functional significance of the observed transcriptional increase remains to be established.

The simultaneous increase in FSTL1, PGC‐1*α*, and VEGF in the CRT group suggests a possible interaction between metabolic and vascular signaling during repeated RT. AMPK acts as an important cellular energy sensor and can promote metabolic adaptation and mitochondrial biogenesis partly through PGC‐1*α* [62]. FSTL1 has also been associated with AMPK activation and eNOS phosphorylation, providing a possible link between metabolic and endothelial responses [6, 60, 63]. In addition, FSTL1 signaling has been linked to TGF‐β/Smad2/3, Akt/mTOR, and ERK pathways that may contribute to vascular remodeling [15, 21, 61, 64–67]. Taken together, these pathways provide a plausible framework in which repeated RT may coordinate metabolic and vascular responses. However, because the present study did not directly measure AMPK phosphorylation, eNOS activity, or these downstream signaling proteins, this pathway should be considered a hypothesis for future investigation rather than a demonstrated mechanism.

In addition to molecular hypoxia‐related pathways, mechanical and hemodynamic stimuli may contribute to the myocardial response to RT. Exercise training can increase coronary transport capacity through structural and functional changes in the coronary microcirculation, including alterations in arteriolar dimensions, vascular reactivity, and capillary exchange capacity [68]. Increased shear stress is also an important stimulus for vascular adaptation [69]. Therefore, the higher VEGF expression observed after CRT may reflect the combined influence of metabolic, mechanical, and hemodynamic stimuli rather than hypoxia alone. This broader interpretation may help explain why VEGF was increased in CRT despite no difference in HIF‐1*α* expression between ART and CRT.

The timing of tissue collection is particularly important when interpreting the present findings. Both ART and CRT cardiac tissues were collected 1 h after the final exercise session. Therefore, the CRT results should not be interpreted as a fully rested basal adaptation to CRT. Rather, they represent an early transcriptional response to the final exercise bout in animals that had undergone chronic training. Exercise‐induced gene expression is highly time‐dependent, with previous studies showing that angiogenic transcripts can change at 0–4 h, with some angiogenic transcripts remaining elevated until 24 h [11, 70, 71], but return to baseline within 24 h after the last session [72]. Similarly, cardiac studies have demonstrated time‐dependent changes in HIF‐1*α* and VEGF expression following myocardial stress [73]. Thus, the present comparison between ART and CRT primarily reflects differences in the early postexercise molecular response between untrained and chronically trained animals. The findings should therefore be interpreted as evidence of both exercise‐induced and training‐associated molecular responses rather than as a direct comparison of acute exercise effects with resting chronic adaptations.

From a translational perspective, the present findings suggest that RT may influence myocardial vascular and metabolic signaling through several interconnected pathways. The increase in HIF‐1*α* and FSTL1 after both ART and CRT, together with the additional increase in PGC‐1*α* and VEGF after CRT, suggests that repeated resistance exercise may produce a broader molecular response involving hypoxia‐related, metabolic, and angiogenic signaling. These findings are broadly consistent with human evidence showing that resistance exercise can modulate angiogenic signaling, including HIF‐1 and VEGF‐related responses, although circulating biomarkers cannot be directly equated with myocardial responses [12]. Resistance exercise is also increasingly recognized as an important component of cardiovascular health promotion, with evidence supporting beneficial effects on cardiovascular risk factors and physiological adaptation [1]. The potential involvement of PGC‐1*α* in the chronic response provides a plausible explanation for the concurrent increase in PGC‐1*α* and VEGF observed in the CRT group, as PGC‐1*α* can regulate VEGF and angiogenic signaling through mechanisms that may complement HIF signaling [19]. More broadly, cardiovascular adaptation to exercise involves coordinated changes in cardiac structure, vascular regulation, and metabolic capacity [74]. However, translation of these findings from healthy rats to humans requires further investigation. Future studies should combine gene expression with protein‐level measurements, myocardial capillary density, vascular function, and relevant signaling pathways to determine whether these transcriptional responses are associated with structural and functional cardiac adaptation.

### 4.1. Strength and Limitation

Several limitations should be considered when interpreting these findings. First, the study assessed mRNA expression without corresponding protein‐level measurements; therefore, changes in transcript abundance cannot be assumed to represent changes in protein activity or downstream signaling. Second, myocardial angiogenesis was not directly assessed using structural or functional measures such as capillary density, endothelial markers, myocardial perfusion, or vascular function. Third, the panel of genes examined was limited and did not allow comprehensive characterization of the signaling networks potentially involved in the observed responses. Fourth, no pathway‐specific interventions were performed; therefore, the present findings cannot establish causal relationships between HIF‐1*α*, PGC‐1*α*, FSTL1, and VEGF. Finally, because cardiac tissue in the CRT group was collected 1 h after the final training session, the observed CRT profile may reflect both adaptations associated with repeated training and the early transcriptional response to the final exercise bout. Future studies incorporating a posttraining washout period, multiple postexercise sampling time points, protein‐level validation, and structural or functional measures of myocardial vascular adaptation are warranted.

## 5. Conclusion

In conclusion, ART and CRT were associated with distinct changes in myocardial expression of angiogenesis‐related genes. ART increased HIF‐1*α* and FSTL‐1 mRNA expression, which may reflect an early transcriptional response to the metabolic and oxygen demands of exercise. In contrast, the higher expression of PGC‐1*α* and VEGF following CRT may reflect a more sustained transcriptional response associated with repeated exercise exposure and metabolic adaptation. Together, these findings suggest that ART and CRT may engage different transcriptional responses related to myocardial angiogenic signaling. However, because this study assessed mRNA expression only, these findings do not establish myocardial angiogenesis or confirm HIF‐1α–dependent or independent mechanisms. Future studies incorporating protein‐level analyses, myocardial capillary density, and vascular functional assessments are needed to determine whether these transcriptional changes translate into structural and functional angiogenic adaptations.

## Author Contributions

Conceptualization: P.K., N.S., and S.S.; methodology: P.K., N.S., and S.S.; software: P.K. and A.M.R.; validation: H.G. and M.R.A.A.S.; formal analysis: P.K., N.S., and A.M.R.; investigation: H.G., S.S., and M.R.A.A.S.; resources: P.K. and S.S.; data curation: P.K., N.S., and S.S.; writing–original draft preparation: P.K. and N.S.; writing–review and editing: S.S., A.M.R., H.G., and M.R.A.A.S.; visualization: P.K.; supervision: S.S., A.M.R., H.G., and M.R.A.A.S.

## Funding

This work was funded by the Indonesian Ministry of Higher Education, Science, and Technology (Grant no. 1662/UN6.3.1/PT/00.2025) awarded to Setiawan Setiawan and Putri Karisa.

## Disclosure

All authors have read and agreed to the published version of the manuscript.

## Ethics Statement

The study protocol was approved by the Ethics Commission of Universitas Padjadjaran (No.1030/UN6.KEP/EC/2024) after obtaining the required permit for the research and was performed following the guidelines for the use of laboratory animals in accordance with AARIVE guidelines.

## Consent

The authors have nothing to report.

## Conflicts of Interest

The authors declare no conflicts of interest.

## Supporting Information

Additional supporting information can be found online in the Supporting Information section.

## Supporting information


**Supporting Information** (1) Supporting Table S1: ARRIVE Guidelines checklist (ARRIVE) 2.0. (2) Supporting Figure S1: GAPDH housekeeping gene expression of each group.

## Data Availability

Data are available on request from the authors.
