# Supplementary material for: Acute and Chronic Resistance Training Differentially Modulate Myocardial Angiogenesis–Related Gene Expression in Wistar Rats
Source: Cardiol Res Pract. 2026 Oct 18;2026:2976039. doi: 10.1155/crp/2976039 (PMC13677139; doi:10.1155/crp/2976039)
Supplement: Supplementary file 1 [file CRP-2026-2976039-s001.zip › Supplementary File 2_GAPDH.docx]

Supplementary File 2

Figure S1. Myocardial GAPDH mRNA expression in sedentary control (CON), acute resistance training (ART), and chronic resistance training (CRT) groups. GAPDH expression levels were comparable across all groups, with no significant differences detected by one-way ANOVA or Tukey’s post hoc test (ns, P>0.05). Data are presented as mean ± SD (n = 8 per group), indicating that GAPDH expression remained stable under the present experimental conditions and is suitable as a reference gene for normalisation of target mRNA levels.
